# Insight into Essential and Complex Autism Spectrum Disorders: Clinical Characteristics, Chromosomal Microarray Analysis, and Risk Factors

**DOI:** 10.3390/genes17091045

**Published:** 2026-08-30

**Authors:** Beyhan Tüysüz, Evrim Çifçi Sunamak, Gizem Durcan, Birol Öztürk, Dilek Uludağ Alkaya, Hazal Cansu Çulpan, Mehmet Barış Korkmaz, Burak Doğangün, Ertuğrul Kıykım

**Affiliations:** 1 Department of Pediatric Genetics, Cerrahpaşa Medical Faculty, Istanbul University-Cerrahpaşa, Istanbul 34098, Türkiye; evrimcifci@gmail.com (E.Ç.S.); drbirolozturk@gmail.com (B.Ö.); dilek.uludagalkaya@iuc.edu.tr (D.U.A.); 2 Department of Pediatrics, Medical Faculty, Atlas University, Istanbul 34158, Türkiye; 3 Department of Child and Adolescent Psychiatry, Cerrahpasa Medical Faculty, Istanbul University-Cerrahpasa, Istanbul 34098, Türkiye; gizem.durcan@iuc.edu.tr (G.D.); burak.dogangun@iuc.edu.tr (B.D.); 4 Department of Public Health, Cerrahpasa Medical Faculty, Istanbul University-Cerrahpasa, Istanbul 34098, Türkiye; hazalcansu.acar@iuc.edu.tr; 5 Department of Neurology, Cerrahpasa Medical Faculty, Istanbul University-Cerrahpasa, Istanbul 34098, Türkiye; bkorkmaz@iuc.edu.tr; 6 Department of Pediatric Nutrition and Metabolism, Cerrahpasa Medical Faculty, Istanbul University-Cerrahpasa, Istanbul 34098, Türkiye; ertugrul.kiykim@iuc.edu.tr

**Keywords:** autism spectrum disease, copy number variations, genes, risk factors, essential phenotype, complex phenotype

## Abstract

**Background/Objectives:** Autism spectrum disorder (ASD) can present with either an essential or a complex phenotype. The aim of this study was to compare clinical characteristics and the diagnostic yield of copy number variations (CNVs) in essential and complex phenotypes, and to evaluate risk factors. **Methods:** A total of 163 Turkish children (126 boys, 37 girls) who met the DSM-5 diagnostic criteria for ASD were evaluated. Chromosomal microarray analysis was performed. **Results:** Among the patients, 21.5% had a complex phenotype and 78.5% had an essential phenotype. Overall, 13.7% of the patients had a developmental/intelligence quotient (DQ/IQ) below 50, most of whom had a complex phenotype. In contrast, 15.3% of the patients had a DQ/IQ of 70 or higher, all of whom had an essential phenotype. The frequency of verbal individuals was 30.5% and did not differ between the two phenotypes. Pathogenic CNVs were identified in 7.4%; 17.1% of the complex group and 4.7% of the essential group. CNVs of uncertain significance that were potentially causal because they included an ASD-associated gene were present in 12.3% of individuals. CNV positivity was significantly higher in individuals with an IQ below 50; interestingly, it was similar between the verbal and non-verbal groups. Besides ultra-rare CNVs, recurrent CNVs associated with ASD were identified. A novel pathogenic CNV was identified at 2q13.33, including *NPHP1* and *BUB1*, both of which are expressed in the brain and are potentially associated with ASD. Advanced parental age and preterm birth were identified as possible risk factors. **Conclusions:** Deep phenotyping is important for the management of both essential and complex phenotypes and allows the identification of patients with a higher probability of having CNVs. Reporting novel or rare CNVs contributes to clarifying the pathogenesis of ASD.

## 1. Introduction

Autism spectrum disorder (ASD) comprises a broad range of neurodevelopmental disorders characterized by deficits in emotional and communication skills as well as intellectual disability, speech delay, and repetitive behaviors [1,2]. The prevalence of ASD in 8-year-old children has been estimated at 18.5 per 1000 [3]. ASD is more common in males than in females, with a ratio of 4–5:1, and exhibits complex inheritance involving both environmental and genetic factors [1,2,4]. Although the marked increase in ASD prevalence in recent years has been attributed to improved diagnostic tests, there is ongoing debate as to whether this is also due to increased awareness of the disorder and the influence of environmental factors [4]. It has been reported that the risk of ASD increases in children whose fathers are over 40 years old at the time of the child’s birth, and in premature or low birth weight children [5,6]. The Simons Foundation Autism Research Initiative (SFARI) Gene database (https://gene.sfari.org), which is continually updated and focused on candidate genes for autism susceptibility, currently lists approximately 1277 human genes associated with ASD.

Over the last 20 years, with the increasing contributions of genetic testing to ASD, the term complex phenotype or syndromic ASD, which refers to a subset of individuals with ASD, has been widely used in clinical practice. Miles et al. [7,8] developed the “Autism Dysmorphology Measure” to describe specific dysmorphic markers in children with autism and classified ASD as complex or essential. Patients with six or more minor malformations and/or at least one major malformation were classified as having complex ASD. Patients with fewer than six minor malformations and no major malformations were classified as having essential ASD. The complex phenotype includes 20–30% of individuals with ASD and has a male/female ratio of 2.5:1, while individuals with essential ASD have more relatives with ASD or neurodevelopmental disorders and a higher male-to-female ratio [9,10]. Several common disorders, including fragile X syndrome (21–50%), Rett syndrome (25–97%), tuberous sclerosis (60%), neurofibromatosis type 1 (13–29%), Down syndrome (5–41%), and inherited metabolic diseases (1–3%), are associated with a high prevalence of ASD [11,12].

Patients with ASD are diagnosed with chromosomal disorders in 3–5%, rare copy number variations (CNVs) in 8–20%, and single-gene disorders in 8–14% [13,14,15]. Chromosomal microarray analysis (CMA) for the detection of CNVs is recommended as a first-line test for patients with ASD [16]. Rare CNVs identified by CMA also provide important information about the complex genetics underlying ASD by highlighting specific regions containing potential genes that predispose individuals to ASD [17,18,19]. A higher rate of pathogenic CNVs has been identified in patients with a complex ASD phenotype [9,10]. Identifying a causative CNV in patients with ASD can reveal previously unnoticed findings related to the phenotype caused by this CNV and guide appropriate management and treatment. The aim of this study is to identify causative CNVs using the CMA technique, to compare clinical findings and CNV positivity in essential and complex ASD, and to investigate risk factors.

## 2. Materials and Methods

### 2.1. Patients

A total of 163 children with ASD, aged between 13 months and 13 years 8 months (median age: 4 years 8 months), with normal karyotype and fragile X analysis, were included in the study. All participants received a clinical diagnosis of ASD according to the criteria of the Diagnostic and Statistical Manual of Mental Disorders (DSM-5), made by child psychiatrists (GD, BD, and MBK) [20]. Children were examined by BT, ECS, and DUA, experienced clinical geneticists; essential and complex phenotypes were defined. Patients with clinically recognizable common genetic syndromes known to be associated with ASD and metabolic diseases were excluded.

Patients were divided into two groups, essential and complex, according to the number of minor and major malformations using the “Development and validation of a measure of dysmorphology: useful for autism sub-group classification” scale [7,8]. Children with six or more minor malformations and/or any major malformations were defined as complex phenotype, and those with fewer than six minor malformations and no accompanying major malformations were defined as having an essential phenotype. Minor malformations are morphological differences that can vary according to ethnicity (with a prevalence of less than 4% in the general population) and do not cause functional impairment (such as upslanted palpebral fissure or a transverse palmar crease). Major malformations also cause functional impairment (such as cleft lip and palate or congenital heart anomalies) [21].

Language ability was assessed by the clinician through observation. A total of 108 children over the age of 4 years were evaluated; those who could use 0–5 words were classified as non-verbal, those with 6–30 functional words and/or two-word phrases were classified as minimally verbal, while the others were considered verbal.

Cognitive and developmental abilities were assessed in 131 children; the remaining 32 children could not be assessed because of noncompliance and significant behavioral problems (Table 1). The developmental quotient (DQ) of 106 children under 6 years of age was measured using the Denver Developmental Test, and the intelligence quotient (IQ) of 25 children over 6 years of age was measured using the Wechsler Intelligence Scales. Developmental delay (DD) or intellectual disability (ID) was classified according to conventional thresholds: DQ/IQ 70–84 (borderline), 50–69 (mild), and below 50 (moderate to severe).

The increased risk of ASD was calculated for maternal and paternal ages at conception in the 30–40, 40–50, and over 50-year age groups, compared with those aged 30 years or younger. The values obtained in this study were compared with those of the general Turkish population (https://www.tuik.gov.tr, accessed on 8 June 2026 and https://www.hsgm.saglik.gov.tr, accessed on 8 June 2026).

Standard deviation scores (SDS) for all anthropometric measurements were calculated using national standards (https://www.ceddcozum.com, accessed on 8 June 2026). Routine biochemical tests, quantitative plasma amino acid and urinary organic acid analyses, and whole blood acylcarnitine profiles via tandem mass spectrometry were performed. Brain magnetic resonance imaging (MRI) and electroencephalography (EEG) were performed in 67 and 70 patients, respectively, among those with epilepsy, macrocephaly, or a complex phenotype.

In this study, data collection and sampling were carried out with prior written informed consent, and all children’s parents or guardians provided written informed consent. Furthermore, this study was approved by the Ethics Committee of Istanbul University-Cerrahpaşa.

### 2.2. Chromosomal Microarray Analysis

After genomic DNA was extracted from peripheral blood leukocytes, CMA was performed in 163 patients. Parental segregation testing was recommended for the families of 75 patients with identified variants, but only 54 families were tested. Samples could not be obtained from the remaining 21 families for various reasons. Two platforms were used to detect CNVs: array-based comparative genomic hybridization (aCGH) for 38 patients (23%) tested in the initial years of the study, and single nucleotide polymorphism array (SNPa) for 125 patients tested later. aCGH was performed using the Agilent SurePrint G3 CGH + SNP Microarray Kit (4 × 180 K) (Agilent Technologies, Santa Clara, CA, USA). Signals were captured with a Roche NimbleGen MS 200 Microarray Scanner (Roche NimbleGen Inc., Madison, WI, USA). Microarray data were analyzed using Feature Extraction software v12.2 and Agilent CytoGenomics v4.0.3.12 software. SNPa was performed using the Illumina iScan CytoSNP-12 v2.1 300K array platform (Illumina, San Diego, CA, USA). The SNP-array results were assessed using BlueFuse Multi version 4.5 (Illumina, San Diego, CA, USA).

#### CNV Classification of Pathogenicity and Interpretation

Genomic positions were reported according to the Human Genome Reference Consortium build GRCh38 (hg38). The pathogenicity of identified CNVs was evaluated using the Varsome and Franklin-Genoox databases, based on American College of Medical Genetics criteria [22]. Pathogenic (P), likely pathogenic (LP) CNVs and variants of uncertain significance (VUS) were searched in the ClinGen (https://www.clinicalgenome.org/, accessed on 8 June 2026) and DECIPHER (https://decipher.sanger.ac.uk/, accessed on 8 June 2026) databases, and published phenotypes of overlapping CNVs were compared with those of the patients. The medical literature was reviewed to clarify the biological functions of selected genes located in CNVs and to determine whether they were associated with ASD. In addition, these genes were categorized according to their association with ASD in the SFARI Gene database as 1 (high confidence), 2 (strong candidate), 3 (proposed evidence), 4 (minimal evidence), 5 (hypothesized), 6 (unsupported), and S (syndromic). If CNVs classified as VUS contained genes associated with autism in the OMIM and SFARI gene databases, they were classified as causative VUS (C-VUS). Along with P-CNVs, they were examined in detail as part of the CNV-positive group.

### 2.3. Statistical Analysis

Statistical analysis was performed using SPSS version 25 (IBM Corp., Armonk, NY, USA). Categorical variables were presented as frequencies and percentages. Parameters including possible risk factors, clinical characteristics, and diagnostic rates of CMA between groups with essential and complex ASD were compared using chi-square and Fisher’s exact tests for categorical variables. To calculate odds ratios (ORs) and 95% confidence intervals (CIs) for the risk associated with increasing parental age, risk estimates were used, with the 20–30-year age group taken as the reference. *p*-values were calculated using the Z-test. A *p*-value < 0.05 was considered significant.

## 3. Results

Of the 163 children, 37 (22.7%) were girls and 126 (77.3%) were boys. The female-to-male ratio was 1:3.4. Metabolic evaluation (quantitative plasma amino acid, urinary organic acid, and whole blood acylcarnitine analyses) revealed that none of the children had a hereditary metabolic disease.

### 3.1. Clinical Features and CNV Positivity in Patients with Essential and Complex ASD

Among the patients, 21.5% had the complex phenotype and 78.5% had the essential phenotype. Table 1 compares clinical features and diagnostic yield between essential and complex phenotypes. The male-to-female ratio was 3.7 in the essential phenotype and 2.5 in the complex phenotype. The rate of affected family members and macrocephaly was higher in the essential group, but this difference was not statistically significant. The seizure rate was significantly higher in the complex phenotype. Of the 70 patients who underwent EEG due to suspected seizures, 12.8% showed abnormal findings, which were more frequent in the complex group than in the essential group. Cranial MRI was performed in 67 patients with epilepsy, macrocephaly, or microcephaly. Minor abnormalities, such as a thin corpus callosum, mildly delayed myelination, or ventricular enlargement, were observed in 20.9% and were significantly more frequent in the complex group.

The frequency of pathogenic CNVs in the essential and complex phenotypes was 4.7% and 17.1%, respectively, and was significantly higher in the complex phenotype (Table 1).

The DQ/IQ scores were significantly lower in the complex group, and CNV positivity was significantly higher in those with scores below 50 compared with those with scores above 50 (Table 2). Of the patients, 30.5% were verbal, 23.2% were non-verbal, and 46.3% were minimally verbal. The frequency of non-verbal patients was significantly higher in the complex group, while the proportions of minimally verbal and verbal patients were similar in the essential and complex groups. CNV positivity was similar across these groups.

### 3.2. Distribution of CMA-Identified Variants According to Pathogenicity and Comparison of Two Different CMA Analysis Platforms

**Figure 1:**
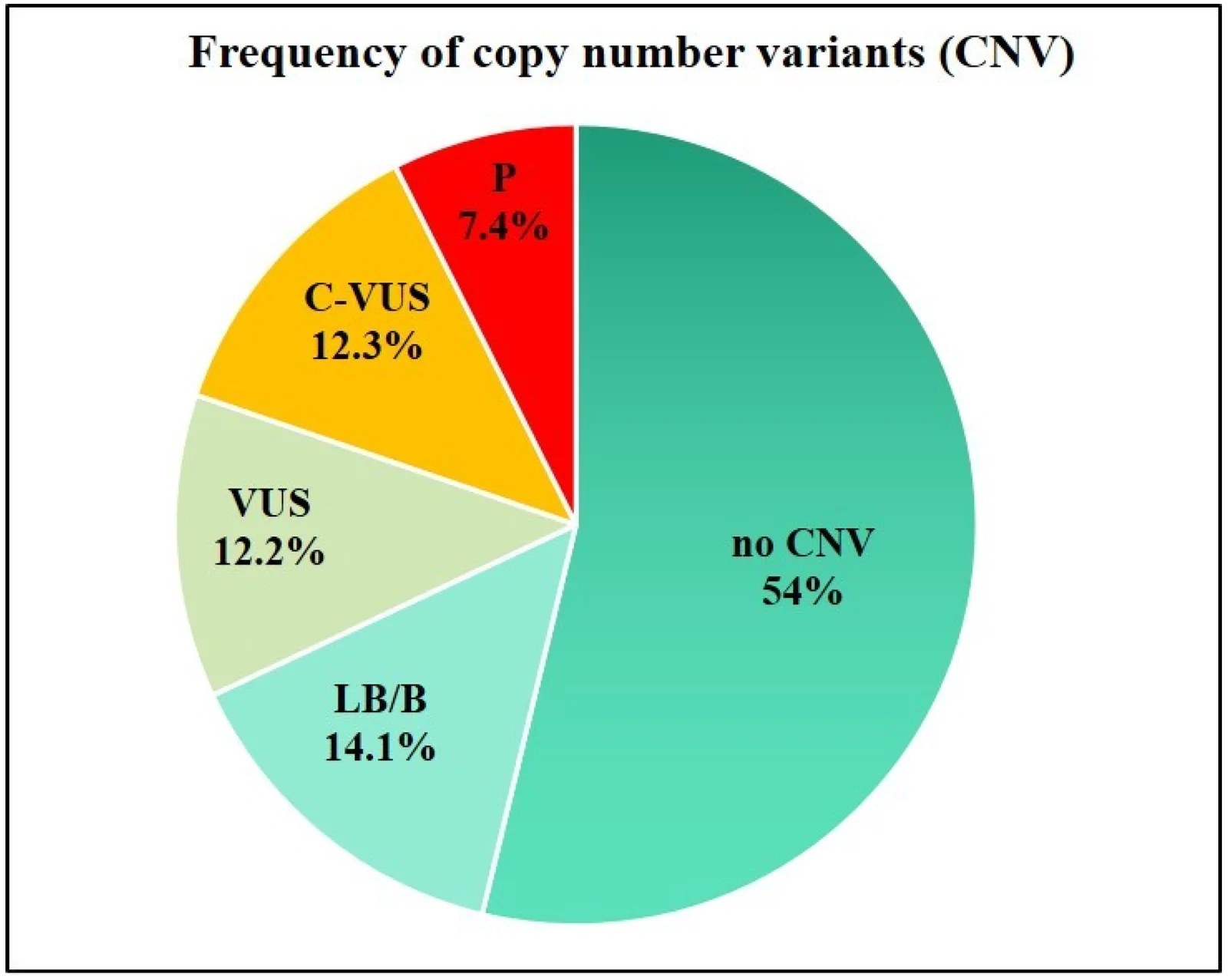
P: Pathogenic, VUS: Variant of uncertain significance, C-VUS: Causative VUS, LB/B: Likely benign/benign.

Seventy-five (46%) of 163 patients were found to have CNVs by CMA. According to pathogenicity classification, 12 (7.4%) were pathogenic, 40 (24.5%) were VUS, and 23 (14.1%) were LB/B variants. Twenty patients (12.3%) with VUS were categorized as having C-VUS (Figure 1). Including patients with P-CNV and C-VUS CNVs, the overall positive CNV rate was 19.6%.

Supplementary Table S1 shows that analysis based on two different microarray platforms revealed no significant difference in pathogenic CNV detection rates between aCGH and SNP-array (5.3% vs. 8.0%; *p* = 0.734). In contrast, C-VUS CNVs were detected significantly more frequently by SNP-array than by aCGH (15.2% vs. 2.6%, respectively; *p* = 0.046). Consequently, the combined diagnostic yield (pathogenic and C-VUS CNVs) was 23.2% for SNP-array and 7.9% for aCGH (*p* = 0.038). To assess whether the higher C-VUS detection rate with SNP-array was related to the detection of smaller CNVs, CNV sizes were compared between platforms. Among pathogenic and C-VUS CNVs combined, the median CNV size was 0.534 Mb (range, 0.310–1.400 Mb) in the aCGH group and 0.533 Mb (range, 0.107–6.600 Mb) in the SNP-array group, with no significant difference between platforms (*p* = 1.000).

Forty-three individuals had one CNV and thirty-two individuals had more than one CNV (VUS or LB/B variants). In 32 patients with multiple CNVs, CMA analysis of the parents revealed that all CNVs were familial.

### 3.3. The Molecular and Phenotypic Characteristics of Pathogenic and C-VUS CNVs

Table 3 shows 12 pathogenic CNVs in 12 patients; three were *de novo*. Six patients had a complex phenotype, and six had an essential phenotype. Three of these CNVs were localized to 22q13.33 and ranged in size from 1 to 6.1 Mb. The other eight were localized to 1p36.33, 2p16.3, 5q35.3, 6p21.31, 9q34.13, 15q11.2, 16p11.2, and 20q13.3 and ranged in size from 111 Kb to 6.6 Mb. Additionally, one pathogenic CNV was a novel 536 Kb deletion at 2q13.33 in a child with an essential phenotype, inherited from a healthy mother.

The characteristics of the C-VUS variants identified in 20 patients are presented in Table 4. Four of these variants were localized in the recurrent CNV regions 15q11.2 and 16p11.2. In addition, three recurrent microduplications involving Xp22.33 were identified in three patients, while a microduplication involving Xq21.1 was detected in one patient. Three patients (P30–P32) had ultra-rare CNVs. Of these, one was classified as Category 3 in the SFARI database, whereas the other two were not listed in SFARI but had been reported in the literature.

CNV segregation could be tested only in the parents of 54 of the 75 patients in whom a variant was identified. Among the parents of 22 patients with P-CNVs and C-VUS CNVs who underwent CMA, 15 CNVs were found to be inherited: nine maternally and six paternally. In Table 3 and Table 4, CNVs are indicated as inherited or de novo where applicable. When the CNVs in Table 3 and Table 4, both *de novo* and familial, were investigated in “Supplementary Table S2” to determine whether they were related to complex or essential phenotypes, *de novo* CNVs were more frequent in patients with a complex phenotype than in those with an essential phenotype (42.9% vs. 26.7%), whereas parental CNVs were observed in 57.1% and 73.3%, respectively; however, the association between inheritance pattern and clinical phenotype was not statistically significant.

### 3.4. Comparison of the Possible Risk Factors in This Cohort and General Turkish Populations

We evaluated risk factors in this cohort with the general Turkish population (Table 5). The association between parental age and ASD risk was assessed using 10-year age categories, with odds ratios (ORs) for each category relative to the 20–29-year age group. A positive association was observed between advanced parental age and ASD risk. Both paternal and maternal age over 40 years were associated with increased ASD risk, with the strongest association observed for paternal age over 50 years. The rate of premature births was significantly higher in the ASD cohort, while the rate of low birth weight was lower.

## 4. Discussion

The aim of this study was to compare clinical characteristics in patients with essential and complex phenotypes, and to investigate the presence of CNVs in both groups. ASD shows variable phenotypic severity and has historically been distinguished by the presence or absence of additional symptoms, called syndromic and non-syndromic or idiopathic autism. Advances in molecular genetic technologies have led to a better understanding of the pathophysiology of autism, with most studies on syndromic autism providing insight into the phenotypic and molecular heterogeneity of neurodevelopmental disorders and suggesting how the study of these disorders can help elucidate the disease mechanisms involved in non-syndromic autism [40,41]. Miles et al. [7,8], after classifying syndromic cases as complex phenotype ASD and others as essential ASD, showed in subsequent studies that 20% of autistic children have complex autism [9,10]. In our study, the rate of complex phenotype was 21.5%, while the rate of essential phenotype was 78.5%, similar to rates reported in the literature (20–30% for complex phenotype) [9,10]. The male-to-female ratio has been reported to be higher in individuals with essential type autism compared with those with the complex phenotype [9,10]. Consistent with the literature, the male/female ratio was 3.7/1 in the essential group and 2.5/1 in the complex group in our study.

CMA studies of children with ASD who had normal chromosome analysis and fragile X syndrome testing found P/LP CNVs in 8–15% of cases [9,10,13,14,17,18,19,24,32,42,43,44]. In the present study, the P-CNV rate was 7.4%, and the rate of C-VUS, which we considered a potential cause of ASD, was 12.3%. The frequency of pathogenic CNVs in the complex ASD phenotype (12.8%, 14.3%, 24.5%, and 29%) has been reported to be higher than in the essential group (3%, 4.2%, 7.3%, and 9%) [9,10,14,45]. The P-CNV ratio was also significantly higher (17.1%) in the complex group than in the essential group (4.7%) in the present study.

In this study, we aimed to compare the developmental abilities of children with complex autism and essential autism, and we obtained cognitive assessments from 131 patients. Overall, 13.7% of the patients had a DQ/IQ below 50, 72% of whom were in the complex phenotype, and 15.3% had a DQ/IQ of 70 or higher, all in the essential phenotype. Approximately 48% of individuals with ASD have an intellectual disability, and 32% experience a loss of previously acquired skills [42,46]. One study found that 14.8% of patients had cognitive abilities within the normal range, with 90% of these in the essential phenotype [9]. Another study reported that 29% of ASD patients had a DQ/IQ below 55, mostly within the complex phenotype [10]. The presence of pathogenic CNVs has been reported to correlate with communication and language deficits in ASD [19,47]. However, recent studies did not find a difference between IQ and CNV positivity in ASD cohorts [9,45]. In the present study, causative CNVs (P-CNVs and C-VUS CNVs) were detected in 38.9% of those with an IQ below 50 and 15% of those with an IQ above 50; this difference was statistically significant. This may be because the majority of patients with DD/ID have a complex phenotype with significantly higher rates of P-CNVs.

There is no consensus on how to assess speech in children with ASD. One study found that 48% of ASD patients over age 8 were verbal [7]. The higher verbal rate at age 8 may be related to increased speech ability with age. Another study found that 34% of those over age 4 were verbal [9]. In the present study, based on clinical observations, when speech development was assessed in those over age 4, 30.5% of patients were verbal. The proportion of non-verbal patients was also higher in the complex group, while the frequency of minimally verbal and verbal patients was the same in both groups. Annunziata et al. [9] suggested that cognitive and verbal abilities were not related to genetic data, whereas another study on the essential phenotype found that the rate of disease-causing CNVs was higher in non-verbal patients [45].

Seizures, EEG abnormalities, and minor brain abnormalities are more common in children diagnosed with complex ASD, whereas macrocephaly is more frequent in the essential phenotype [9,10,19]. In our study, the rate of macrocephaly was higher in the essential phenotype, but this difference was not significant; however, in the complex phenotype, the rates of seizures, EEG abnormalities, and brain abnormalities were significantly higher than in patients with the essential phenotype.

Seven of the 12 patients in whom we found pathogenic CNVs had recurrent CNVs involving genes well known in ASD (*SHANK3*, *SYNGAP1*, *NRXN1*, *NSD1,* and *SAMD11*), six of whom had complex phenotypes [23,48,49,50,51,52]. Two of the other five patients with the essential phenotype carried rare CNVs, *POMT1* and *SOX18*, which are listed as associated with ASD [25,53]. We also identified a novel pathogenic 536 kb deletion at 2q13.33 in a child with the essential phenotype; this CNV was inherited from a healthy mother. To date, neither this CNV nor the genes it encompasses, *NPHP1* (MIM:609583) and *BUB1* (MIM:620183), have been shown to be associated with ASD. *NPHP1* is a “Joubert syndrome 4” gene involved in actin and microtubule complex structure, cell signaling, and division; *BUB1* is a primary microcephaly gene involved in mitosis. Analyses of the protein functions encoded by genes associated with ASD highlight their association with chromatin modelling and neuronal growth [48,49]. It is possible that these two genes and the novel CNV they contain are associated with ASD.

Two pathogenic CNVs and four C-VUSs in chromosomal regions 15q11.2 and 16p11.2, which are known to harbor the most common (4–5%) recurrent CNVs associated with ASD, were also identified in this study. These regions include ASD-related genes such as *UBE3A*, *NIPA1*, *ATP10A*, *TAOK2,* and *HERC2*, and CNVs involving these genes were identified in six patients with an essential phenotype similar to that reported in the other patients (Table 3 and Table 4) [30,31,32,53,54]. Both regions are rich in homologous low-copy repeats that can cause chromosomal rearrangements via non-allelic homologous recombination during meiosis [53,54].

In the C-VUS group presented here (Table 4), five of the 20 patients had the complex phenotype, and 15 had the essential phenotype. Three cases with the essential phenotype had recurrent Xp22.33 microduplication and one case with the complex phenotype had ATRX-containing Xq21.1 microduplication [26,30,31]. Nine other patients had CNVs encompassing genes classified as category 2 in the SFARI database, as well as one of three patients with the essential phenotype, an ultra-rare CNV encompassing *FBXL13* (category 3), reported in only one case, and two others had CNVs containing genes not yet included in the SFARI database, but each reported in a single study. The first CNV included *NRG3*, a schizophrenia susceptibility gene, while the second included *SETDB2*, which encodes a histone methyltransferase associated with ASD and contains a methyl-CpG binding domain and a SeT domain [27,28,29,33,34,35,36,37,38].

The recurrence rate of ASD is higher in families with multiple cases of ASD or neurodevelopmental disorders, particularly in the essential phenotype, and it has been suggested that this may be because ASD patients share a biology with primary neurodevelopmental disorders presenting with features such as attention deficit/hyperactivity disorder or epilepsy [9,10]. In our study, the rate of familial ASD and related disease was 17.2% (Table 1). Although the number of affected individuals in each family was higher in the essential phenotype group than in the complex group, the difference was not significant. We also examined the significance of the frequencies of inherited and de novo CNVs shown in Table 3 and Table 4, and in the essential and complex phenotypes in Supplementary Table S2, but no significant difference was found between the two groups. The fact that familial CNVs did not show a more severe complex ASD phenotype may be related to factors such as incomplete penetrance and variable expression [48,49].

Furthermore, it has been hypothesized that two or more CNVs, most of them inherited from the parents, may increase susceptibility to ASD, that patients with these CNVs may develop a more severe phenotype, and that neurodevelopmental disorders are more common in these families, consistent with a “double-hit” model [9,19,32,48,49]. In this study, we found multiple CNVs in 32 subjects, all of which were familial. Since all of these CNVs were non-causal VUSs or benign variants, we therefore considered it unlikely that they would positively contribute to the double hit model.

We also investigated familial and perinatal risk factors such as whether advanced parental age, preterm birth, and low birth weight are predisposing factors for ASD. When we compared fathers’ ages at the time of their children’s birth with those of the Turkish population, the proportion of fathers over 40 was three times higher than that of fathers under 30, and the proportion of fathers over 50 was four times higher. Similarly, mothers’ ages at the time of their children’s birth were twice as high for those over 30 compared with the population under 30, and four times higher for those over 40. One study found that the probability of children with ASD being born to men aged 40 and over was higher than for children of men under 30, whereas advanced maternal age showed no association with ASD [55]. However, recent studies have reported that both advanced paternal and maternal age increase the risk of autism [56,57].

We found that premature births were more frequent in the ASD group than in the general Turkish population, supporting the hypothesis that prematurity may be a risk factor for ASD. In contrast, the rate of low birth weight was not found to be high in patients with ASD; in fact, it was lower than in the general Turkish population [39].

The main limitations of this study include the short follow-up period for the patients, the inability to cognitively assess all of them, the inability to perform CMA analysis on all parents of patients with CNV, and the lack of further analysis to confirm whether novel and VUS-CNVs are the cause of the disease. In addition, the use of two microarray platforms with different probe densities may have introduced technical heterogeneity. Although pathogenic CNV detection rates were similar between platforms, C-VUS detection was higher with the SNP-array. Therefore, platform-related differences should be considered when interpreting the results.

## 5. Conclusions

This study showed that, in a well-defined ASD cohort, 21.5% of patients exhibited the complex phenotype and 78.5% exhibited the essential phenotype. Pathogenic CNVs were identified in 7.4%. In the complex phenotype group, the proportion of children with an IQ below 50 and those who were non-verbal was significantly higher than in those with the essential phenotype. However, CNV positivity was significantly higher in those with an IQ below 50, while it was similar between verbal and non-verbal groups. By reporting a novel pathogenic CNV associated with ASD, together with ultra-rare CNVs and the ASD-associated genes they encompass, we have strengthened and expanded the pool of ASD-related CNVs. The reporting of novel CNVs contributes to elucidating the pathogenesis of ASD.

## Figures and Tables

**Table 1:** Diagnostic yield and distribution of clinical characteristics in patients according to complex and essential phenotypes.

Clinical Phenotype	Essential	Complex	Total	*p*-Value
**Number**	**128 (78.5%)**	**35 (21.5%)**	**163**	**-**
**Male/Female**	101/27 (3.7/1)	25/10 (2.5/1)	126/37 (3.4/1)	-
**Familiality**	24 (18.7%)	4 (11.4%)	28 (17.2%)	0.309
**Macrocephaly**	15 (11.7%)	2 (5.7%)	17 (10.4%)	0.303
**Seizures**	13 (10.1%)	9 (25.7%)	22 (13.5%)	0.017
**DQ/IQ (*n* = 131)**	***n*** = **110**	***n*** = **21**	***n*** = **131**	
<50	5 (4.5%)	13 (61.9%)	18 (13.7%)	<0.001
50–69	85 (77.3%)	8 (38.1%)	93 (71%)	<0.001
≥70	20 (18.2%)	0	20 (15.3%)	0.042
**Language Level (*n* = 108)**	***n*** = **77**	***n*** = **31**	***n*** = **108**	
Non-verbal	11 (14.3%)	14 (45.2%)	25 (23.2%)	0.001
Minimal verbal	39 (50.6%)	11 (35.5%)	50 (46.3%)	0.201
Verbal	27 (35.1%)	6 (19.3%)	33 (30.5%)	0.109
**EEG Abnormalities (*n* = 70)**	***n*** = **50** 3 (6.0%)	***n*** = **20** 6 (30.0%)	***n*** = **70** 9 (12.8%)	0.007
**Brain MRI Abnormalities (*n* = 67)**	***n*** = **41** 4 (9.7%)	***n*** = **26** 10 (38.5%)	***n*** = **67** 14/67 (20.9%)	0.005
**CNV Diagnostic Yield**	**6 (4.7%)**	**6 (17.1%)**	**12/163 (7.4%)**	**0.022**

CNV: Copy number variant; DQ: Development quotient; EEG: Electroencephalography; IQ: Intelligence quotient; MRI: Magnetic resonance imaging.

**Table 2:** Distribution of DQ/IQ and language level according to chromosomal microarray results.

Patients	Causative CNV			No Causative CNV *	Total	*p*-Value
	P	C-VUS	Subtotal			
**DQ/IQ (*n* = 131)**						
<50	6	1	7 (38.9%)	11 (61.1%)	18 (100%)	0.046 **
50–69	5	10	15 (16.1%)	78 (83.9%)	93 (100%)	
≥70	1	1	2 (10%)	18 (90.0%)	20 (100%)	
**Language Level (*n* = 108)**						
Non-verbal	3	2	5 (20%)	20 (80.0%)	25 (100%)	0.699
Minimal verbal	5	3	8 (16%)	42 (84.0%)	50 (100%)	
Verbal	1	2	3 (9.1%)	30 (90.9%)	33 (100%)	

DQ: Development quotient; IQ: Intelligence quotient; CNV: Copy number variant; P: Pathogenic; C-VUS: Causative variant of uncertain significance; * No causative CNV included non-causal VUS, likely benign, benign variants, and cases with no CNV. ** The *p*-value was calculated by comparing the DQ/IQ < 50 and ≥50 groups.

**Table 3:** Characteristics of pathogenic copy number variations (CNVs) in 12 patients.

Patient No	Age/ Sex	aCGH /SNPa	Cytogenetic Location (Loss or Gain)	CNV Size (Genomic Coordinates)	Inherited	ASD-Related Genes	OMIM Number or Reference/SFARI Database Category	Clinical Phenotype
1	2.5 years/ Female	SNPa	**22q13.33** (deletion)	**6.1 Mb** arr[GRCh38] (44,603,124_50,734,546)x1	NA	*SHANK3, NCAPH2*	606232/ *SHANK3*: Category 1 *NCAPH2:*Category 3	Complex
2	1.5 years/ Male	SNPa	**22q13.33** (deletion)	**6.05 Mb** arr[GRCh38] (44,703,945_50,759,411)x1	NA	*SHANK3,* *NCAPH2*	606232/ *SHANK3*:Category 1 *NCAPH2:*Category 3	Complex
3	13.8 years/ Male	SNPa	**22q13.33** (deletion)	**1 MB** arr[GRCh38] (49,730,445_50,759,410)x1	*De novo*	*SHANK3, NCAPH2*	606232/ *SHANK3*:Category 1 *NCAPH2:*Category 3	Complex
4	4.9 years/ Female	SNPa	**6p21.31** (deletion)	**111 Kb** arr[GRCh38] (33,430,085_33,541,164)x1	Maternal	*SYNGAP1*	612621/ Category1	Complex
5	13 months/ Male	aCGH	**5q35.2q35.3** (deletion)	**1.4 Mb** arr[GRCh38] (176,323,394_177,709,289)x1	*De novo*	*NSD1* *UIMC1*	613638/ *NSD1*: Category 1 *UIMC1:* Category 2	Complex
6	11.8 years/ Male	SNPa	**1p36.33** (deletion)	**2.36 Mb** arr[GRCh38] (82,154_2,512,975)x1	NA	*SAMD11*	[23] Category 2	Complex
7	9.8 years/ Female	SNPa	**2p16.3** (deletion)	**225 Kb** arr[GRCh38] (50,579,019_50,804,140)x1	Maternal	*NRXN1*	614332/ Category 1	Essential
8	6.5 years/ Male	SNPa	**9q34.13** (duplication)	**519 Kb** arr[GRCh38] (131,160,527_131,680,020)x3	*De novo*	*POMT1*	[24] Category 2	Essential
9	6.2 years/ Male	SNPa	**20q13.3** (deletion)	**253 Kb** arr[GRCh38] (63,946,260_64,200,172)x1	Paternal	*SOX18*	[25] There is no SFARI database	Essential
10	2.1 years/ Female	SNPa	**2q13.33** (deletion)	**536.6 Kb** arr[GRCh38] (110,102,095_110,638,682)x1	Maternal	*NPHP1* *BUB1*	Novel	Essential
11	4 years/ Female	SNPa	**15q11.2q13.2** (duplication)	**6.6 Mb** arr[GRCh38] (23,416,359_30,061,288)x3	NA	*UBE3A* *ATP10A* *HERC2*	608636/ *UBE3A* Category 1 *ATP10A* Category 2 *HERC2* Category S	Essential
12	4.2 years/ Female	aCGH	**16p11.2** (deletion)	**533.8 Kb** arr[GRCh38] (29,645,363_30,179,247)x1	Maternal	*TAOK2*	611913/ Category2	Essential

aCGH: array-based comparative genomic hybridization, SNPa: Single nucleotide polymorphism array, CNV: Copy number variant, ASD: Autism spectrum disease, NA: Not available.

**Table 4:** Characteristics of causative variants of uncertain significance (C-VUS) copy number variations (CNVs) associated with autism spectrum disease in 20 patients.

Patient No	Age/ Sex	aCGH /SNPa	Cytogenetic Location (Loss or Gain)	CNV Size (Genomic Coordinates)	Inherited	ASD-Related Genes	OMIM Number Or Reference/SFARI Database Category	Clinical Phenotype
13	8.5 years/ Male	SNPa	**Xq21.1** (duplication)	**500 Kb** arr[GRCh38] (77,071,854_77,572,257)x2	*De novo*	*ATRX*	300032, [26] Categories 1 and S	Complex
14	10 years/ Male	SNPa	**17q21.31** (deletion)	**387 Kb** arr[GRCh38] (46,170,855_46,557,630)x1	Paternal	*KANSL1*	610676, [27] Categories 1 and S	Complex
15	2.2 years /Female	aCGH	**5p11.2** (duplication)	**310 Kb** arr[GRCh38] (11,505,204_11,815,139)x3	Maternal	*CTNND2*	[28] Category 2	Complex
16	5.1 years/ Male	SNPa	**19p12** (deletion)	**829 Kb** arr[GRCh38] (20,051,886_20,881,230)x1	NA	*ZNF626*	Category 2	Complex
17	8 years/ Male	SNPa	**20p12.1** (deletion)	**307 Kb** arr[GRCh38] (14,783,801_15,090,053)x1	Paternal	*MACROD2*	[29] Category 2	Complex
18	2.5 years/ Male	SNPa	**15q11.2** (deletion)	**2.8 Mb** arr[GRCh38] (19,956,119_22,786,139)x1	NA	*NIPA1*	[24] Category2	Essential
19	8.2 years/ Male	SNPa	**15q11.2** (duplication)	**436 Kb** arr[GRCh38] (22,666,554_23,102,646)x3	Paternal	*NIPA1*	[24] Category 2	Essential
20	3.8 years/ male	SNPa	**16p11.2** (duplication)	**1.4 Mb** arr[GRCh38] (32,491,547_33,802,728)x3	NA	-	Autism, susceptibility 14B (614671)	Essential
21	6.8 years/ Male	SNPa	**16p11.2** (duplication)	**1.5 Mb** arr[GRCh38] (32,418,652_33,994,428)x3	NA	-	Autism, susceptibility 14B (614671)	Essential
22	5.8 years/ Male	SNPa	**Xp22.33** (duplication)	**659 Kb** arr[GRCh38] (43,118_702,706)x2	Maternal	*SHOX*	[30] Category 2	Essential
23	3 years/ Male	SNPa	**Xp22.33** (duplication)	**449 Kb** arr[GRCh38] (1,309,080_1,758,112)x2	Maternal	*ASMT*	[31] Category 2	Essential
24	3 years/ Male	SNPa	**Xp22.33** (duplication)	**445 Kb** arr[GRCh38] (1,312,643_1,758,112)x2	NA	*ASMT*	[31] Category 2	Essential
25	5.5 years/ Male	SNPa	**10q21.3** (deletion)	**218 Kb** arr[GRCh38] (66,508,523_66,726,616)x1	*De novo*	*CTNNA3*	[32] Category 2	Essential
26	7 years/ Male	SNPa	**5q35.2** (duplication)	**508 Kb** arr[GRCh38] (175,021,746_175,529,464)x3	Paternal	*DRD1*	[33] Category 2	Essential
27	7 years/ Male	SNPa	**11q14.3** **(deletion)**	**353 Kb** arr[GRCh38] (88,772,308_89,125,673)x1	Paternal	*GRM5*	[34] Category 2	Essential
28	7.5 years/ Male	SNPa	**2q31.2q31.3** (duplication)	**932 Kb** arr[GRCh38] (179,118,845_180,050,166)x3	*De novo*	*ZNF385B*	Category 2	Essential
29	9.5 years/ Male	SNPa	**13q12.12** (duplication)	**1.3Mb** arr[GRCh38] (22,975,645_24,286,102)x3	NA	*SACS*	[35] Category 2	Essential
30	8 years/ Male	SNPa	**7q22.1** (deletion)	**432kb** arr[GRCh38] (102,467,705_102,899,802)x1	*De novo*	*FBXL13*	[36] Category 3	Essential
31	6.3 years/ Male	SNPa	**10q23.1** (deletion)	**107Kb** arr[GRCh38] (82,227,959_82,335,015)x1	Maternal	*NRG3*	[37] There is no SFARI database	Essential
32	4.9 years/ Male	SNPa	**13q14.2** (duplication)	**232 Kb** arr[GRCh38] (49,400,479_49,633,084)x3	Maternal	*SETDB2*	[38] There is no SFARI database	Essential

aCGH: array-based comparative genomic hybridization, SNPa: Single nucleotide polymorphism array, CNV: copy number variant, ASD: autism spectrum disorder, Category S (syndromic), NA: Not available.

**Table 5:** Comparison of the frequency of risk factors associated with ASD with the general Turkish population.

	This Cohort *n* = 163	Turkish Population (Annual Live-Birth) * n = 1,237,172	Possible Risk	Odds Ratio (OR) (95% Confidence Interval-CI)	*p*-Value
**Paternal age**					
20–29 **	30 (18.4%)	468,471 (37.9%)	6.4:100,000	1	
30–39	104 (63.8%)	629,730 (50.9%)	16.5:100,000	2.58 (1.72–3.87)	<0.001
40–49	26 (16.0%)	129,030 (10.4%)	20.2:100,000	3.15 (1.86–5.32)	<0.001
≥50	3 (1.8%)	9941 (0.8%)	30.2:100,000	4.71 (1.44–15.45)	<0.011
**Maternal age**					
20–29 **	58 (35.6%)	710,687 (57.4%)	8.2:100,000	1	
30–39	91 (55.8%)	490,051 (39.7%)	18.6:100,000	2.28 (1.64–3.16)	<0.001
40–49	14 (8.6%)	36,434 (2.9%)	38.4:100,000	4.71 (2.63–8.44)	<0.001
**Preterm birth**	30 (18.4%)	136,867 (11%) ***	21.9:100,000	1.67 (1.11–2.51)	<0.003
**Low birth weight**	5 (3%)	99,800 (8%) ***	5.0:100,000	0.36 (0.15–0.88)	<0.025

* http://www.tuik.gov.tr, accessed on 8 June 2026, ** reference group, *** https://www.hsgm.saglik.gov.tr, accessed on 8 June 2026 [39].

## Data Availability

The data that support the findings of this study are available from the corresponding author upon reasonable request.

## Supplementary Materials

The following supporting information can be downloaded at: https://www.mdpi.com/article/10.3390/genes17091045/s1, Table S1: Comparison of two different microarray platform-based analyzes; Table S2: Association between CNV inheritance pattern and clinical phenotype.

## Abbreviations

The following abbreviations are used in this manuscript:

ASD	Autism Spectrum Disorder
B/LB	Likely benign/Benign
CMA	Chromosomal microarray analysis
CNV	Copy number variant
DD	Developmental delay
DQ	Development quotient
EEG	Electroencephalography
ID	Intellectual disability
IQ	Intelligence quotient
MRI	Magnetic resonance imaging
P/LP	Pathogenic/Likely pathogenic
SDS	Standard deviation score
SFARI	Simons Foundation Autism Research Initiative database
C-VUS	Causative variant of uncertain significance
VUS	Variant of uncertain significance
