# Supplementary material for: Insight into Essential and Complex Autism Spectrum Disorders: Clinical Characteristics, Chromosomal Microarray Analysis, and Risk Factors
Source: Genes (Basel). 2026 Aug 30;17(9):1045. doi: 10.3390/genes17091045 (PMC13606228; doi:10.3390/genes17091045)
Supplement: Supplementary file 1 [file genes-17-01045-s001.zip › genes-4494771-supplementary.pdf]

Supplementary Table S1. Comparison of two different microarray platform-based analyzes

Results	aCGH (n=38)	SNP array (n=125)	Total (n=163)	P-value
Pathogenic	2 (5.3%)	10 (8.0%)	12 (7.4%)	0.734
Causative VUS	1 (2.6%)	19 (15.2%)	20 (12.3%)	0.046
P-CNV and C-VUS CNV	3 (7.9%)	29 (23.2%)	32 (19.6%)	0.038
Non-causative VUS	4 (10.5%)	16 (12.8%)	20 (12.3%)	1.00
LB/B varyant	2 (5.3%)	21 (16.8%)	23 (14.1%)	0.073
Negative	29 (76.3%)	59 (47.2%)	88 (54.0%)	0.002

Supplementary Table S2. Association between CNV inheritance pattern and clinical phenotype

Inheritance	Complex n (%)	Essential n (%)	P-value
**De novo**	3 (42.9)	4 (26.7)	0.630
**Parental**	4 (57.1)	11 (73.3)	
**Total**	**7**	**15**	
